# Comparison of the Intestinal Microbial Community in Ducks Reared Differently through High-Throughput Sequencing

**DOI:** 10.1155/2019/9015054

**Published:** 2019-03-10

**Authors:** Yan Zhao, Kun Li, Houqiang Luo, Longchuan Duan, Caixia Wei, Meng Wang, Junjie Jin, Suzhen Liu, Khalid Mehmood, Muhammad Shahzad

**Affiliations:** ^1^College of Animal Science, Wenzhou Vocational College of Science and Technology, Wenzhou 325006, China; ^2^College of Veterinary Medicine, Huazhong Agricultural University, Wuhan 430070, China; ^3^Department of Pathobiology, College of Veterinary Medicine, University of Illinois at Urbana-Champaign, USA; ^4^University College of Veterinary & Animal Sciences, The Islamia University of Bahawalpur, 63100, Pakistan

## Abstract

Birds are an important source of fecal contamination in environment. Many of diseases are spread through water contamination caused by poultry droppings. A study was conducted to compare the intestinal microbial structure of Shaoxing ducks with and without water. Thirty 1-day-old Shaoxing ducks (Qingke No. 3) were randomly divided into two groups; one group had free access to water (CC), while the other one was restricted from water (CT). After 8 months of breeding, caecal samples of 10 birds from each group were obtained on ice for high-throughput sequencing. A total of 1507978 valid sequences were examined and clustered into 1815 operational taxonomic units (OTUs). At phylum level, Firmicutes (41.37%), Bacteroidetes (33.26%), Proteobacteria (13.67%), and Actinobacteria (8.26%) were found to dominate the microbial community in CC birds, while Firmicutes (53.62%), Bacteroidetes (33.06%), and Actinobacteria (11.13%) were uncovered to be the prime phyla in CT ducks. At genus level, Bacteroides (25.02%), Escherichia-Shigella (11.02%), Peptococcus (7.73%) and Parabacteroides (5.86%) were revealed to be the mainly genera in the CC group ducks, while Bacteroides (18.11%), Erysipelatoclostridium (10.94%), Ruminococcaceae_unclassified (10.43%), Lachnospiraceae_unclassified (5.26%), Coriobacteriales_unclassified (5.89%), and Faecalibacterium (4.2%) were detected to staple the microbial flora in the CT birds. One phylum and 13 genera were found to have the significant difference between the two bird groups (p<0.05). At phylum level, Proteobacteria in CT ducks were found to be obviously lower than ducks in CC birds (p<0.05). At genus level, Escherichia-Shigella (p<0.05) and Peptococcus (p<0.05) were found to be notably lower in CT birds, while Erysipelatoclostridium (p<0.05), Ruminococcaceae_unclassified (p<0.01), Coriobacteriales_unclassified (p<0.05), Faecalibacterium (p<0.01), Atopobiaceae_unclassified (p<0.01), Alistipes (p<0.05), Eggerthellaceae_unclassified (p<0.05), Prevotella_7 (<0.05), Rikenellaceae_RC9_gut_group (p<0.05), Prevotellaceae_uncultured (p<0.05), and Shuttleworthia (p<0.05) were observed to be prominently higher in CT ducks. In conclusion, the present study revealed the effects of keeping ducks away from swimming with obvious changes in the microbial community. Though higher microbial richness was found in the ducks without swimming, more pathogenic genera including Eggerthella, Erysipelatoclostridium, Alistipes, Prevotella_7, and Shuttleworthia; zoonotic genera including Eggerthella and Shuttleworthia; inflammatory genus Alistipes; anti-inflammatory Faecalibacterium genus; and tumor genus Rikenellaceae were examined in these ducks. The CT ducks also showed significant changes at genera level regarding the metabolism (Peptococcus, Ruminococcaceae, and Coriobacteriales).

## 1. Introduction

Poultry is of great importance for economic and human nutrition accommodation. The poultry in China accounts for about 25% of the world population and approximately 40% of fowl in Asia [1]. Four common types of ducks are reared in China: Gaoyou duck, Peking duck, Shaoxing duck, and Weishan sheldrakes. These ducks contribute greatly to the Chinese poultry industry. Shaoxing ducks are commonly bred in more than 10 provinces of the country. However, an unavoidable water contamination caused by these ducks has always been causing trouble to local population [2]. Keeping the birds away from water for swimming seems to be indeed an effective and undemanding method to drop water contamination.

The human and animal complicated microbial community was made up of over thousands of microbial genera [2]. Those numerous microorganisms are of great importance for acting the role of gastrointestinal system protection, synthesizing and metabolizing parts of NHVs (nutrients, hormones, and vitamins), eliminating drugs and toxic metabolites, inducting and regulating host responses to various pathogens, protecting from pathogens, and helping with the development and maturation of immune cells [3–7]. The changes or disturbances of microbial flora were found to have relationships with various gastrointestinal diseases, which may cause impaired digestive, diminished weight gain, being even mortal to animals [5]. Infectious diseases may cause serious disaster for animal health and productivity in developing countries [8–10]. However, scarce information is available regarding taking the ducks out of pool causing effects on the microbial community of the birds. Therefore, we performed this study to reveal the microbial community changes by comparing the microbial structure of Shaoxing ducks with and without water for swimming through high-throughput sequencing.

## 2. Materials and Methods

### 2.1. Ethics

All animal experiments and procedures were conducted under the relevant procedures of Proclamation of the Standing Committee of College of Animal Sciences, Wenzhou Vocational College of Science and Technology, Wenzhou, People's Republic of China.

### 2.2. Birds Management and Caeca Sampling

**Figure 1:**
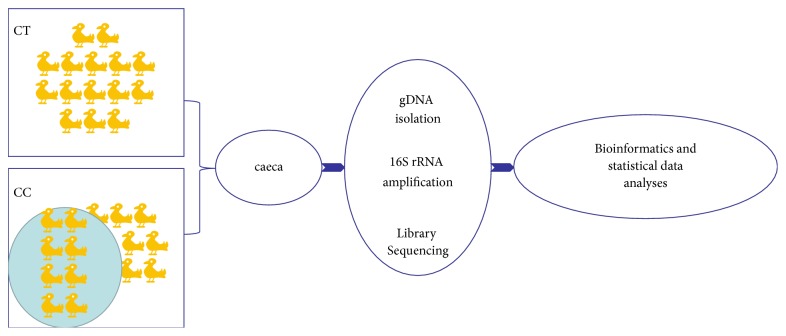
Flow diagram of the current research design. The ducks in CC and CT groups were bred differently as the ducks of CC group had free access to water.

In current study, a total of 30 1-day-old Shaoxing ducks (Qingke No. 3) were reared and bred in a local duck farm in Cangnan County, Wenzhou, China. The ducks were randomly divided into two groups (CC and CT) (Figure 1). The ducks in CC group (CC1-CC10) and CT group (CT1-CT10) were bred with commercial diets and normal drinking water as previously reported [11]. Birds in CC group had free access to water for swimming, while ducks in CT group were confined without water. After 8 months of breeding, 10 caecal samples from each group were obtained and frozen in liquid nitrogen immediately. All the samples were stored at −80°C for further analysis.

### 2.3. DNA Isolation and Gene Amplification

The microbial genomic DNA from each duck sample was isolated by employing the commercial QIAamp® Fast DNA Mini Kits (Qiagen Ltd., Germany) in accordance with manufacturer's specification, as described in previous study [4]. The gene of 16S rRNA (V3-V4 variable region) was amplified with primers (F: ACTCCTACGGGAGGCAGCAG and R: GGACTACHVGGGTWTCTAAT) [12]. The PCR mixture including 18 ul autoclaved distilled water, 10 ul PCR Buffer (5×), 4.5 ul dNTPs (2.5 mM), 10 ul GC Buffer (5×), 4 ul DNA Template, 0.5 ul Taq E, and 1.5 ul of each forward and reverse primer (working concentration: 10 uM) in a 50 ul reaction volume was prepared. Each of 35 PCR cycles consisted of 98°C for 15s, 55°C for 30s, and 72°C for 27s after an initial hot start at 98°C for 3 min and ending with 72°C for 10 min. All current DNA amplification products were examined by 1.5% agarose gel stained with ethidium bromide; DNA bands were recovered using AxyPrep DNA Gel Recovery Kit (Axygen Biosciences, USA).

### 2.4. Library Preparation and Sequencing

Libraries were constructed by utilizing NEB Next Ultra DNA Library Prep Kit for Illumina (NEB, USA) following the explanatory memorandum, and index codes were added as described in previous study [6, 13]. All the library products were quantified utilizing Qubit 2.0 Fluorometer (Thermo Fisher Scientific, US). Then all the amplification libraries were sequenced via high-throughput sequencing on an Illumina MiSeq platform (Illumina, San Diego, US).

### 2.5. Bioinformatics and Statistical Analysis

Quantitative Insights Into Microbial Ecology (QIIME, v1.8.0) was used to remove error or question sequences in the current study [14]. Operational taxonomic units (OTUs) were obtained by using USEARCH (Version 7.1 http://drive5.com/uparse/) via 97% similarity and removing the divergence sequences clustering (<3%) [6, 15]. OTUs were taxonomically analyzed via BLASTn tool against a curated UNITE database [16]. The analysis of Alpha and Beta diversity was done according to previously reported studies [4, 6]. The microflora of birds at phylum and genus levels were identified by employing QIIME [6]. Heat maps were designed based on R package “gplots” [4]. PCA (Principal Component Analysis), PCoA (Principal Coordinate Analysis), NMDS (Nonmetric Multidimensional Scaling), LEfSe (Linear Discriminant Analysis Effect Size), and LDA (Linear Discrimination Analysis) were carried out according to previous research reports [17–19]. To discover the duck group difference at different levels, one-way analysis of variance was used followed by Tukey's honest test for continuous variables, and the differences were considered statistically significant when p < 0.05 through the IBM SPSS Statistics 24.0 (SPSS Somers, NY).

## 3. Results

### 3.1. The Microbial Community Diversities of Ducks in Different Groups

**Figure 2:**
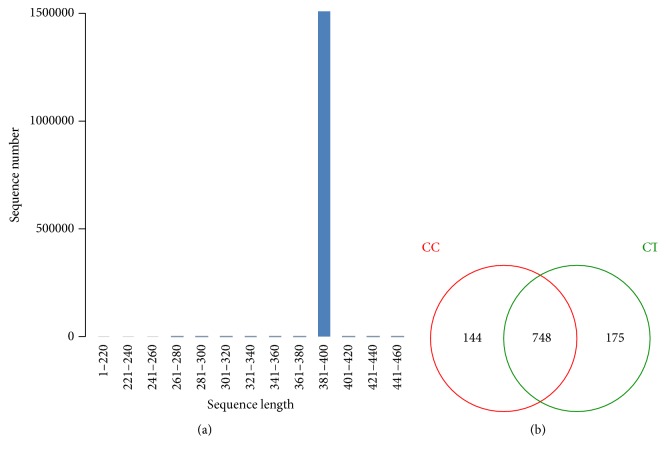
(a) Length distribution of trimmed sequences and (b) OTUs Venn analysis. (a) Length distribution of trimmed sequences. Almost all the sequences from CC and CT groups were about of 381-400 bp. (b) Venn analysis of OTUs from CC and CT groups. Most OTUs were shared by both groups, with 144 and 175 OTUs owned by CC or CT groups. Ducks in CC groups had free access to swimming, while the birds in CT group were confined to land.

**Figure 3:**
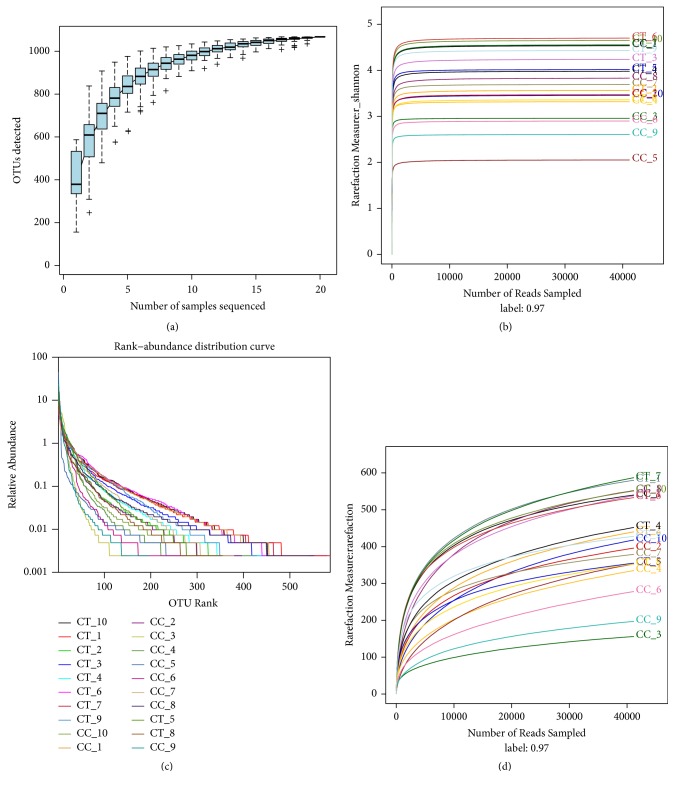
Species richness and diversity analysis of different groups. (a) Species accumulation curves of the current study. (b) The rank abundance curve of all birds in two groups. (c) The Shannon diversity index rarefaction curve of different duck samples in two groups. (d) The rarefaction curve of all the birds in two groups. Ducks in CC (CC1-CC10) groups had free access to swimming, while birds in CT (CT1-CT10) group were limited to land only.

A total of 1507978 valid sequences were examined with 99.92% sequences in the length of 381-400 bp (Figure 2(a)). All those sequences were clustered into 892 and 923 OTUs, respectively, in CC and CT group. A total of 748 OTUs were shared into two groups (Figure 2(b)). The species accumulation curve was found extremely horizontal when samples reached 20, which showed that the selected samples in the current study were reasonable (Figure 3(a)). All the values of coverage were extremely close to 1.00, which demonstrated a considerable high number of libraries detected out in each duck (Table 1). The rank abundance curves of different birds indicated were long and smooth broken line indicating high abundance and uniform distribution of species of the birds (Figure 3(b)) [6]. The low values of Simpson and horizontal broken Shannon curves revealed high diversities of those ducks (Table 1; Figure 3(c)). The community richness index of Chao and ACE different duck samples showed a high richness of microbial flora in each duck, with the gradual rarefaction curves of the birds (Table 1; Figure 3(d)).

### 3.2. The Microbial Community Structure in Different Levels in Different Duck Groups

At phylum level, Firmicutes (41.37%), Bacteroidetes (33.26%), Proteobacteria (13.67%), and Actinobacteria (8.26%) were found to dominate the microbial community in all the CC birds, while Firmicutes (53.62%), Bacteroidetes (33.06%), and Actinobacteria (11.13%) were uncovered to be the prime phyla in CT ducks (Figure 5). At genus level, Bacteroides (25.02%), Escherichia-Shigella (11.02%), Peptococcus (7.73%), and Parabacteroides (5.86%) were revealed to be the main genera in CC group ducks, while Bacteroides (18.11%), Erysipelatoclostridium (10.94%), Ruminococcaceae_unclassified (10.43%), Lachnospiraceae_unclassified (5.26%), Coriobacteriales_unclassified (5.89%), and Faecalibacterium (4.2%) were detected to staple the microbial flora in the CT birds (Figure 6).

### 3.3. Comparison of the Microbial Community Structure in Different Bird Groups

A clear difference between CC and CT duck was revealed by the microbial community bar plot with cluster tree calculated by employing Bray-Curtis (Bray-Curtis Distance Coefficient Bray-Curtis) (Figure 7) [20]. The results of PCA, PCoA, and NMDS all showed a distinct shift of microbial structure between two groups (Figure 8). By employing the Metastats analysis, 1 phylum and 13 genera were found to have significant difference between the two bird groups. At phylum level, Proteobacteria in CT ducks were found to be obviously lower than ducks in CC birds (p=0.0167<0.05) (Figure 9). At genus level, Escherichia-Shigella (p=0.0364) and Peptococcus (p=0.0429) were found to be notably lower in CT birds (p<0.05), while Erysipelatoclostridium (P=0.0407<0.05), Ruminococcaceae_unclassified (p=0.0027<0.01), Coriobacteriales_unclassified (p=0.0235<0.05), Faecalibacterium (p=0.0014<0.01), Atopobiaceae_unclassified (p=0.0093<0.01), Alistipes (p=0.014<0.05), Eggerthellaceae_unclassified (p=0.0128<0.05), Prevotella_7 (p=0.0155<0.05), Rikenellaceae_RC9_gut_group (p=0.0385<0.05), Prevotellaceae_uncultured (p=0.0345<0.05), and Shuttleworthia (p=0.0124<0.05) were observed to be prominently higher in the CT ducks (Figure 10). The heat map in the present results indicated that there was an obvious difference of phylum of Proteobacteria in different groups (Figure 11). Significant difference was also discovered among the genera of Klebsiella, Brevibacterium, Bacillus, Lactococcus, Anaerofustis, Bacteroidales_unclassified, Rikenellaceae_RC9_gut_group, Rikenellaceae_unclassified, Ruminococcus_torques_group, Negativibacillus, Blautia, Ruminococcaceae_UCG−005, Ruminococcaceae_UCG−014, Shuttleworthia, Prevotellaceae_uncultured, and Faecalibacterium in two duck groups (Figure 12). By employing the utilization of LEfSe (http://huttenhower.sph.harvard.edu/galaxy/root?tool_id=lefse_upload) and LDA (linear discriminant analysis), the important microbiome of CC ducks was shown in red color, while that of CT birds was shown in green color (Figure 13). Obvious difference of important microorganism was uncovered with misalignment in red and green color between the two groups.

## 4. Discussion

In China, over 10 billion of poultry each year (National Bureau of Statistics of China, (http://data.stats.gov.cn/adv.htm?m=advquery&cn=C01) mark a great contribution to the economic and food supply of the country. Such an amazing number of birds raise a question mark towards the quality of water for human consumption.

**Figure 4:**
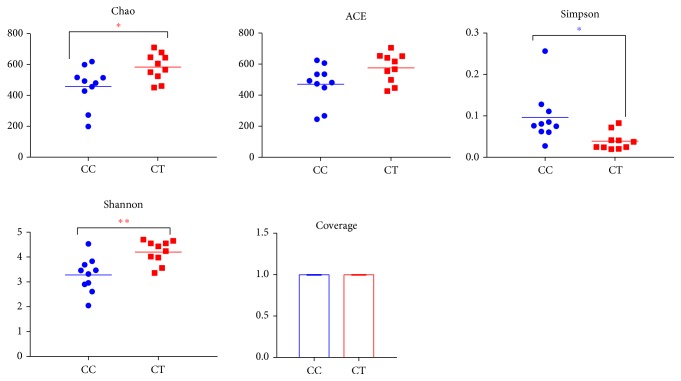
Comparison of the microfloral microbial diversity index (Chao1, ACE, Simpson Shannon, and Coverage) between two duck groups. Alpha-diversity analysis of microbiota of the two groups was compared according to the microfloral microbial diversity index (Chao1, ACE, Simpson Shannon, and Coverage) between two duck groups. Ducks in CC groups had free access to swimming, while birds in CT group were reserved to land (*∗ρ*<0.05; *∗∗ρ*<0.01 for Student's t-test).

**Figure 5:**
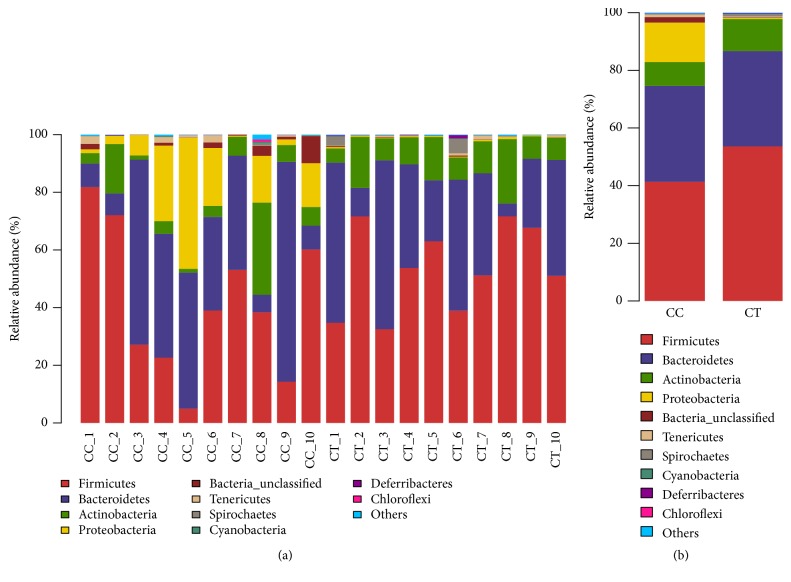
The microbial community structure at phylum level: (a) different ducks; (b) different groups. The microbial community structure difference was compared at phylum level in both groups: (a) comparing the microbial differences in all the ducks; (b) comparing the microbial differences in the two groups. The ducks in CC (CC1-CC10) group had free access to water, and birds in CT (CT1-CT10) group were confined to land.

**Figure 6:**
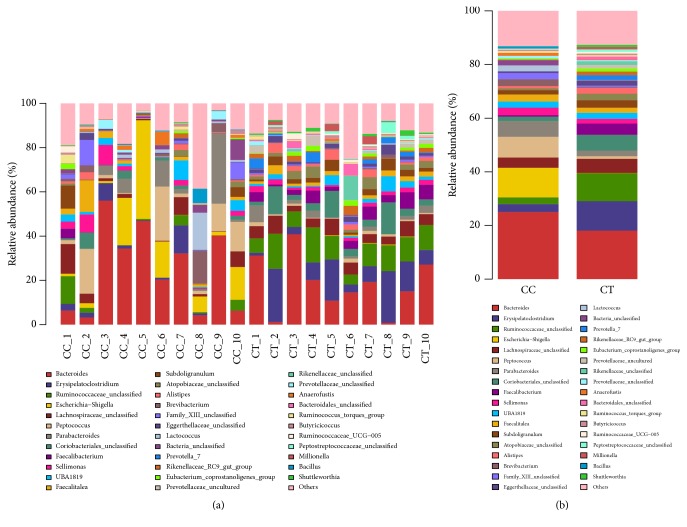
The microbial community structure at genus level: (a) different ducks; (b) different groups. The microbial community structure difference was compared at genus level in CC and CT groups: (a) comparing the microbial differences in all the ducks; (b) comparing the microbial differences in the two groups.

**Figure 7:**
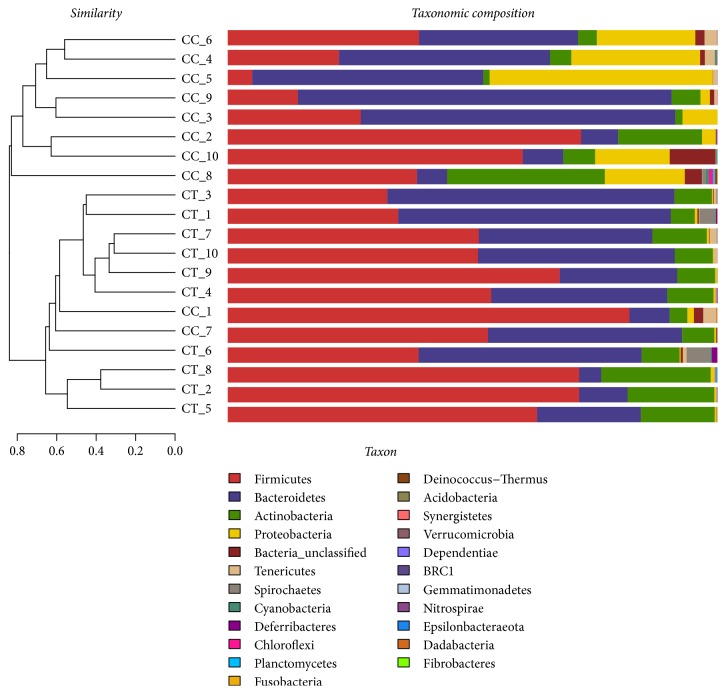
Microbial community bar plot with cluster tree of the ducks.

**Figure 8:**
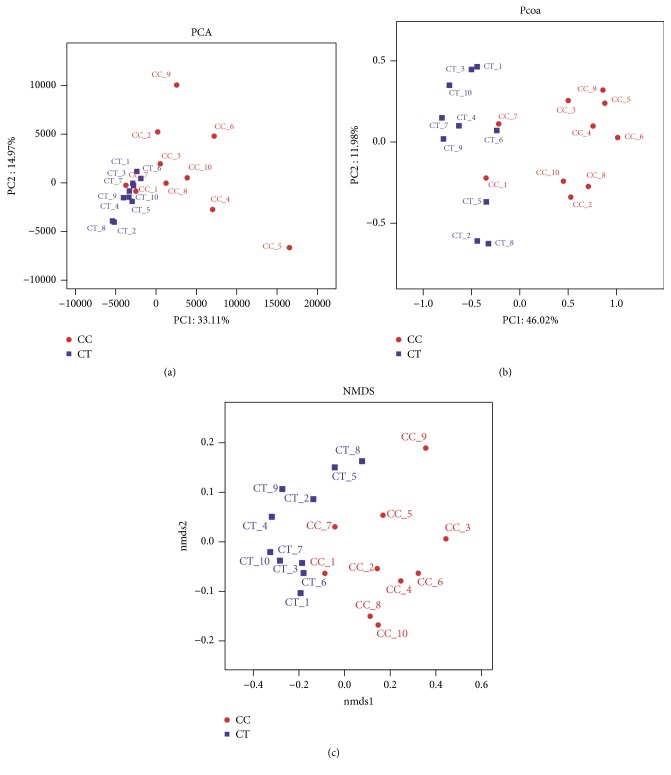
Multiple samples analysis of microbial community of the ducks: (a) PCA; (b) PCoA; (c) NMDS. Multiple samples of microbial community of the ducks were analyzed in CC and CT groups of ducks: (a) Principal Component Analysis (PCA) of microbial community of the ducks in CC and CT groups; (b) Principal Coordinates Analysis (PCoA) of microbial community of the ducks in CC and CT groups; (c) Nonmetric Multidimensional Scaling (NMDS) of microbial community of the ducks in CC and CT groups.

Previously, a study was conducted in Shaoxing ducks, the same ducks used in our study, which described the changes in gut microbiota reared on litter and plastic mesh floor [21]. In the current study, we employed 16S rRNA gene Illumina HiSeq sequencing to compare the intestinal microbiota in CC and CT ducks for the first time. High diversities and richness of microbial flora were found in each duck which was in line with previous studies [4, 6]. The index of Shannon in CT ducks was significantly higher, while Simpson was obviously at higher level (Figure 4), which might be due to the two different statistical techniques, as both methods concern the diversities of microbial community [6]. The Chao index was shown to be conspicuously higher in CT ducks (Figure 8), which was in accordance with previously found decreased richness of microbial community in diarrheal dogs and deer [22, 23]. The present results possibly mean that taking the birds out of water increased the richness of microbial community.

**Figure 9:**
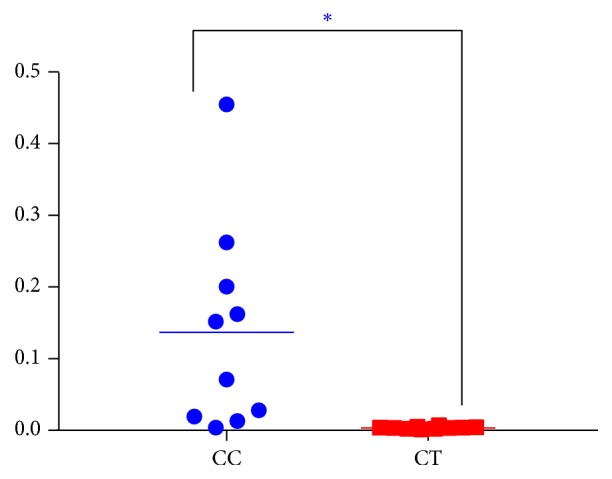
Comparison of the microbial community structure in phylum level in different groups.

**Figure 10:**
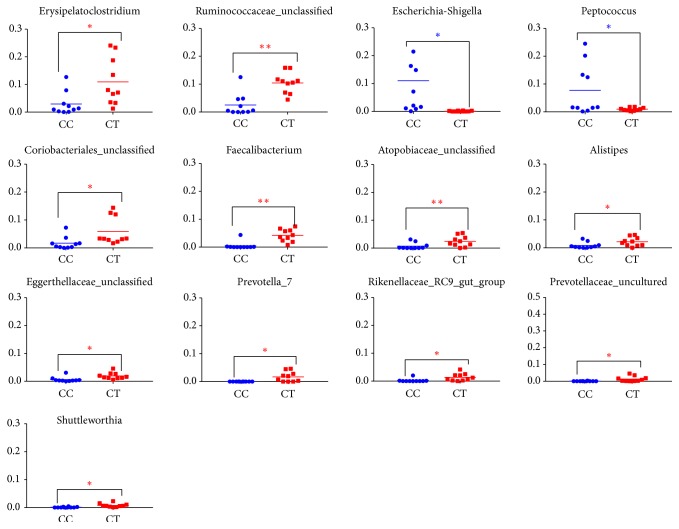
Comparison of the microbial community structure in genus level in different duck groups.

**Figure 11:**
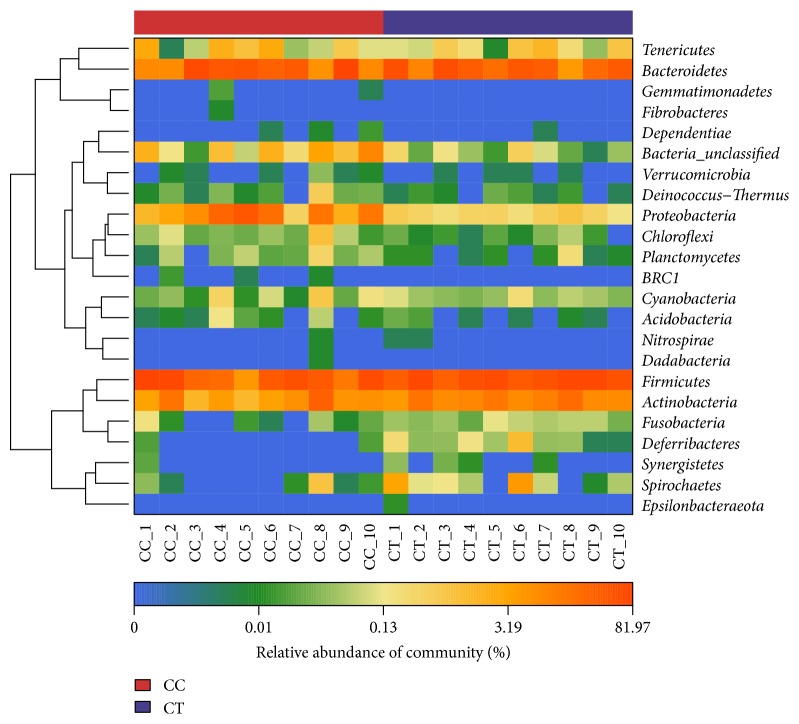
Heat map analysis of 23 most abundant phyla of each bird was carried out in CC (CC1-CC10) group with free access to water and CT (CT1-CT10) group without water.

**Figure 12:**
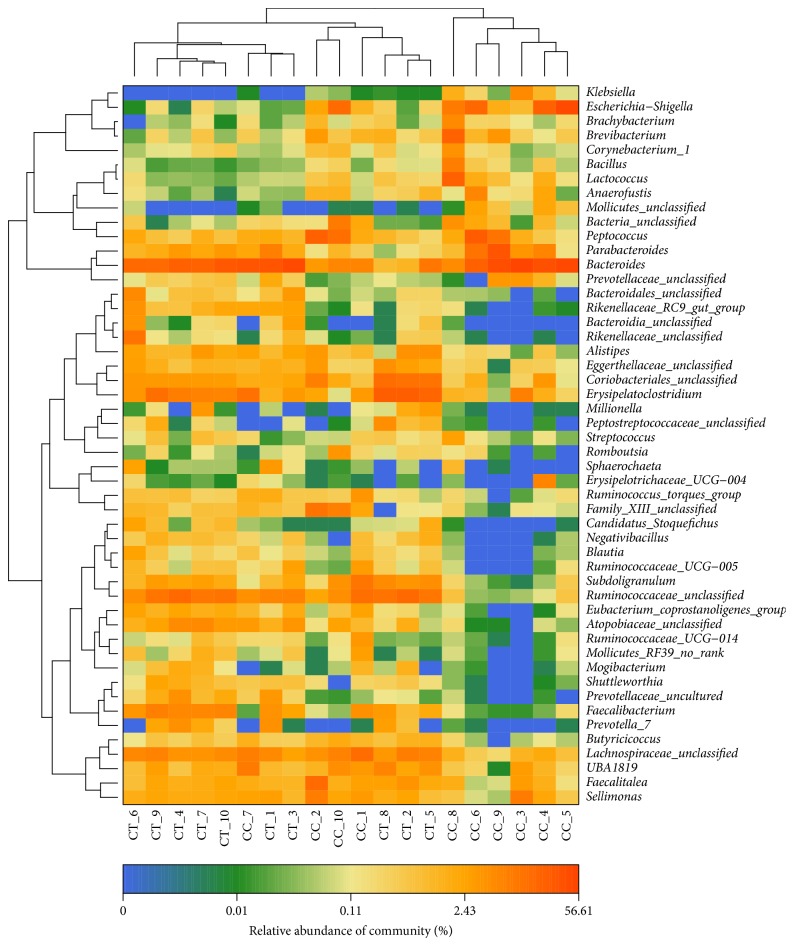
Heat map of 50 most abundant genera of each ducks.

**Figure 13:**
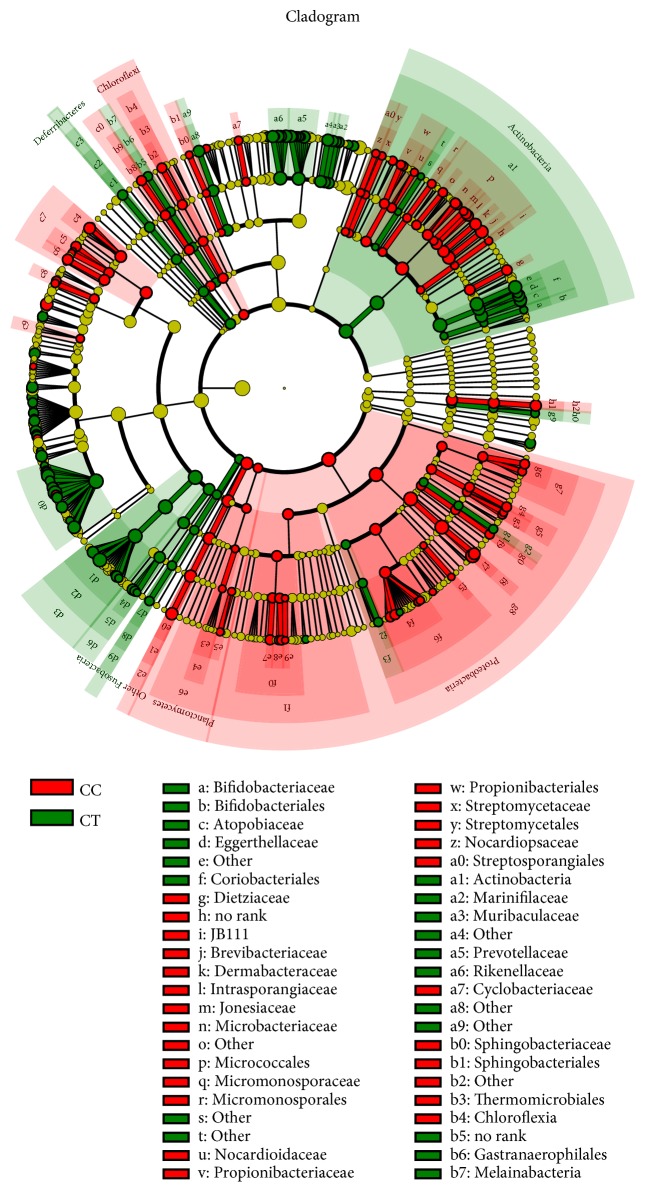
The cladogram analyzed LEfSe of the ducks. The cladogram analysis of LEfSe and LDA (linear discriminant analysis) was done in CC and CT groups of ducks. The important microbiome of CC ducks is shown in red color, while that of CT birds is shown in green color. Ducks in CC groups had free access to swimming while birds in CT group were limited to land.

One phylum and 13 genera in CT birds were observed significantly in CC ducks (Figures 9 and 10). Among 13 genera, 2 genera (Escherichia-Shigella and Peptococcus) were discovered at much lower level in CT group, while 11 genera (Erysipelatoclostridium, Ruminococcaceae_unclassified, Coriobacteriales_unclassified, Faecalibacterium, Atopobiaceae_unclassified, Alistipes, Eggerthellaceae_unclassified, Prevotella_7, Rikenellaceae_RC9_gut_group, Prevotellaceae_uncultured, and Shuttleworthia) were shown to be obviously higher in CT ducks (Figure 9). The commonly known Escherichia-Shigella was found to be associated with a proinflammatory status [24] leading to the inflammation of the colon mucosa [22]. These bacterial pathogens are mostly transmitted through feces, food, and water [23, 24]. The genera of Eggerthella, Erysipelatoclostridium, Alistipes, and Prevotella_7 were considered as opportunistic pathogens [25–27] with Eggerthella having the potential of zoonosis [26]. The genus Shuttleworthia has one known species,* Robinsoniella peoriensis*, which was originally isolated from swine and could infect immune-competent humans [28, 29]. From the results, it is deduced that taking the ducks away from swimming decreased the pathogenetic genus of Escherichia-Shigella; however, more pathogens (Eggerthella, Erysipelatoclostridium, Alistipes, Prevotella_7, and Shuttleworthia) obviously grew in the microbial community. Peptococcus genus was reported to have relationship with respiratory glucose metabolism [30, 31]. Ruminococcaceae is connected to cellulose-degrading capacity [32]. Coriobacteriales are able to metabolize wide variety of carbohydrates and other metabolites [33]. Coriobacteriaceae genus has been observed to increase significantly in the ceca of mice in response to stress [34]. These results are suggestive that keeping the ducks away from swimming can possibly affect the metabolism of birds. Faecalibacterium is involved in anti-inflammatory activity within the gut [34, 35] and Alistipes have been shown to be associated with gut inflammation [36]. Rikenellaceae were found to be higher in tumor syndrome cancer patients [37]. From the changes, we may reveal that swimming can bring about the changes in inflammatory and anti-inflammatory processes and even tumor mechanisms due to changes in microbial flora. Because of the lack of information regarding Atopobiaceae and Rikenellaceae, the relevance of its relationship with two different groups cannot be speculated [38].

In conclusion, the present study for the first time revealed the effects of keeping ducks away from swimming with obvious changes in the microbial community of birds. Although higher microbial richness was found in the ducks without swimming, more pathogenetic genera (Eggerthella, Erysipelatoclostridium, Alistipes, Prevotella_7, and Shuttleworthia), even zoonotic genera (Eggerthella and Shuttleworthia), inflammatory genera (Alistipes), anti-inflammatory genera (Faecalibacterium), and tumor (Rikenellaceae) related genera were examined in the ducks. Also CT ducks showed significant changes in genera regarding the metabolism (Peptococcus, Ruminococcaceae, and Coriobacteriales). The research performed herein may contribute to and highlight the strategic process concerning the water pollution in animal husbandry industry.

## Figures and Tables

**Table 1:** The microflora microbial diversity index (Chao, ACE, Simpson, Shannon, and Coverage) of each duck.

Group	Sample ID	Chao	ACE	Simpson	Shannon	Coverage
CC	1	599	606	0.0274	4.53	0.997910
	2	493	492	0.0761	3.47	0.997618
	3	199	245	0.0748	2.96	0.998882
	4	514	473	0.0852	3.32	0.997229
	5	480	481	0.2565	2.05	0.997059
	6	428	535	0.1108	2.9	0.997424
	7	456	448	0.0807	3.69	0.998226
	8	619	624	0.0606	3.83	0.997497
	9	273	267	0.128	2.61	0.998517
	10	516	534	0.0621	3.46	0.997108
CT	1	647	653	0.0242	4.55	0.997594
	2	566	554	0.0718	3.56	0.997156
	3	678	651	0.0413	4.24	0.997059
	4	550	567	0.041	3.98	0.997327
	5	461	426	0.0375	4.02	0.998080
	6	606	617	0.0196	4.7	0.997715
	7	710	705	0.0249	4.55	0.997011
	8	451	446	0.0827	3.36	0.997813
	9	524	498	0.0246	4.43	0.998056
	10	644	640	0.0204	4.65	0.997570

## Data Availability

The data used to support the findings of this study are available from the corresponding author upon request.
